# Supplementary material for: Proximity proteomics in a marine diatom reveals a putative cell surface-to-chloroplast iron trafficking pathway
Source: eLife. 2021 Feb 16;10:e52770. doi: 10.7554/eLife.52770 (PMC7972479; doi:10.7554/eLife.52770)
Supplement: Transparent reporting form [file elife-52770-transrepform.docx]

***eLife’s* transparent reporting form**

We encourage authors to provide detailed information *within their submission* to facilitate the interpretation and replication of experiments. Authors can upload supporting documentation to indicate the use of appropriate reporting guidelines for health-related research (see [EQUATOR Network](http://www.equator-network.org/%20)), life science research (see the [BioSharing Information Resource](https://biosharing.org/)), or the [ARRIVE guidelines](http://www.plosbiology.org/article/info:doi/10.1371/journal.pbio.1000412) for reporting work involving animal research. Where applicable, authors should refer to any relevant reporting standards documents in this form.

If you have any questions, please consult our Journal Policies and/or contact us: [editorial@elifesciences.org](mailto:editorial@elifesciences.org).

**Sample-size estimation**

- You should state whether an appropriate sample size was computed when the study was being designed
- You should state the statistical method of sample size computation and any required assumptions
- If no explicit power analysis was used, you should describe how you decided what sample (replicate) size (number) to use

Please outline where this information can be found within the submission (e.g., sections or figure legends), or explain why this information doesn’t apply to your submission:

We did not conduct power analysis as part of our experimental design. We followed a standard practice in the field to perform experiments with triplicate or quintuplicate cultures.

**Replicates**

- You should report how often each experiment was performed
- You should include a definition of biological versus technical replication
- The data obtained should be provided and sufficient information should be provided to indicate the number of independent biological and/or technical replicates
- If you encountered any outliers, you should describe how these were handled
- Criteria for exclusion/inclusion of data should be clearly stated
- High-throughput sequence data should be uploaded before submission, with a private link for reviewers provided (these are available from both GEO and ArrayExpress)

Please outline where this information can be found within the submission (e.g., sections or figure legends), or explain why this information doesn’t apply to your submission:

One pTF-APEX2 expressing cell line (s2) was used to inoculate either triplicate (Amplex UltraRed assay: Figure 2 legend, “Amplex UltraRed assay and resorufin imaging” section in Material and Methods; electron microscopy: Figure 3 legend, “Transmission electron microscopy (TEM)” section in Material and Methods) or quintuplicate (proximity proteomics: section corresponding to Figure 4, Figure 4 legend, “Proximity-dependent proteomic mapping” section in Material and Methods) experimental cultures. Flavin reduction assays with purified pTF.CREG1 were conducted in triplicate (Figure 6—figure supplement 2 legend, “Flavin reductions assays” section in Materials and Methods). The raw quantitative mass spectrometry proteomics data upload and access information can be found in the “Additional files” section.

**Statistical reporting**

- Statistical analysis methods should be described and justified
- Raw data should be presented in figures whenever informative to do so (typically when N per group is less than 10)
- For each experiment, you should identify the statistical tests used, exact values of N, definitions of center, methods of multiple test correction, and dispersion and precision measures (e.g., mean, median, SD, SEM, confidence intervals; and, for the major substantive results, a measure of effect size (e.g., Pearson's r, Cohen's d)
- Report exact p-values wherever possible alongside the summary statistics and 95% confidence intervals. These should be reported for all key questions and not only when the p-value is less than 0.05.

Please outline where this information can be found within the submission (e.g., sections or figure legends), or explain why this information doesn’t apply to your submission:

Figure 4—source data 1 (last column) contains P values associated with all the proteins detected in our quantitative mass spectrometry proteomics experiment including the thirty-eight statistically significant proteins (P value ≤0.05) with APEX2/WT ratios of at least 1.5 highlighted in Figure 4B.

(For large datasets, or papers with a very large number of statistical tests, you may upload a single table file with tests, Ns, etc., with reference to sections in the manuscript.)

**Group allocation**

- Indicate how samples were allocated into experimental groups (in the case of clinical studies, please specify allocation to treatment method); if randomization was used, please also state if restricted randomization was applied
- Indicate if masking was used during group allocation, data collection and/or data analysis

Please outline where this information can be found within the submission (e.g., sections or figure legends), or explain why this information doesn’t apply to your submission:

Allocation of samples into experimental groups was based on the absence or presence of the pTF-APEX2 expressing episome in *Phaeodactylum tricornutum* cells (Figure 2D, Figure 3, and Figure 4A–B), or the absence or presence of the pTF.CREG1 protein in the reaction mixture (Figure 6—figure supplement 2C). This is stated in the corresponding sections and figure legends.

**Additional data files (“source data”)**

- We encourage you to upload relevant additional data files, such as numerical data that are represented as a graph in a figure, or as a summary table
- Where provided, these should be in the most useful format, and they can be uploaded as “Source data” files linked to a main figure or table
- Include model definition files including the full list of parameters used
- Include code used for data analysis (e.g., R, MatLab)
- Avoid stating that data files are “available upon request”

Please indicate the figures or tables for which source data files have been provided:

Figure 2, Figure 4, Figure 4—figure supplement 1, Figure 5—figure supplement 1, Figure 6, Figure 6—figure supplement 2, Figure 7, Figure 7—figure supplement 2, Supplemental file 1—Table S1.
